# Seasonality, Weather, and Obstetric Level of Care: An Analysis of Rural Delivery Locations

**DOI:** 10.3390/ijerph23060731

**Published:** 2026-05-30

**Authors:** Andreas Thorsen, Maggie L. Thorsen, Ronald G. McGarvey, Sean Harris

**Affiliations:** 1 Jake Jabs College of Business and Entrepreneurship, Montana State University, Bozeman, MT 59717, USA; sean.harris1@montana.edu; 2 Department of Sociology and Anthropology, Montana State University, Bozeman, MT 59717, USA; margaret.thorsen@montana.edu; 3 IESEG School of Management, Univ. Lille, CNRS, UMR 9221–LEM–Lille Economie Management, F-59000 Lille, France; r.mcgarvey@ieseg.fr

**Keywords:** birth seasonality, obstetric bypassing, obstetric level of care, perinatal care access

## Abstract

**Highlights:**

**Public health relevance—How does this work relate to a public health issue?**

- This research addresses seasonal differences in perinatal care access within rural northern mountainous regions of the US where extreme weather and vast distances create barriers to seeking care.
- The study examines the seasonality of obstetric bypassing, where patients travel past their local hospital to deliver at a more distant facility.

**Public health significance—Why is this work of significance to public health?**

- Montana’s birth volume peaks in June rather than the US late-summer norm, which is important for accurate hospital capacity planning and resource allocation.
- Bypassing to Level 3 sub-specialty hospitals, located in urban areas, increased over the study period, signaling potential sustainability challenges for smaller rural obstetric units.

**Public health implications—What are the key implications or messages for practitioners, policy makers, and/or researchers in public health?**

- Hospitals must manage shifting patient flows, as winter weather conditions are associated with higher patient retention at local Level 1 hospitals offering basic obstetric care, consistent with reduced travel during inclement conditions, while summer conditions lead to surges in bypassing toward Level 3 hospitals.
- Level 1 hospitals lose the most volume during non-winter months and may need targeted retention strategies to recapture the high number of births currently lost to other hospitals.

**Abstract:**

Birth seasonality in the United States typically peaks in late summer, yet research has rarely examined these patterns in northern mountainous regions or across different hospital obstetric levels. This study investigates the temporal dynamics of maternal healthcare-seeking behavior in Montana, specifically focusing on seasonality in birth volume and obstetric bypassing (delivering at a non-local hospital). We conducted a retrospective analysis of 98,524 birth records (2014–2022) at hospitals with Level 1, 2, and 3 obstetric units, integrating driving distances and monthly county-level climate data. Statistical analyses included calculating observed-to-expected (O-E) ratios to identify seasonality and regression models to test interactions between season, hospital level, and weather. Montana birth volume is distinguished by a peak in June and a trough in January, with seasonality most pronounced at Level 3 hospitals. Obstetric bypassing significantly decreases during winter (O-E ratio 0.95), particularly for Level 1 hospitals, while increasing during warm, high-precipitation months. Over time, bypassing toward Level 3 hospitals has risen from 13.6% to 20%. We conclude that seasonality and weather correlates are associated with significant variation in care-seeking patterns, reflecting the unique challenges facing rural hospitals.

## 1. Introduction

### 1.1. Background

Seasonality of births in the United States is distinguished by a peak during August-September and a trough during April-May [1]. Research has shown that the trough is intensified in the southern part of the US [2]. Research indicates that birth seasonality follows a distinct latitudinal gradient, with northern populations often exhibiting peaks that differ significantly from southern regions [3]. There are differences in seasonality across race, education, and marital status [1]. While extreme summer temperatures reduce fertility in most states, Montana has not historically exhibited this pattern [4].

Patients navigate the tension between seeking risk-appropriate care and the systemic constraints of a fragile rural healthcare infrastructure through a healthcare-seeking dynamic called obstetric bypassing [5,6]. Obstetric bypassing refers to the act of delivering at a non-local obstetric unit, which often necessitates increased travel distances for patients. Following the Andersen–Aday model, this behavior is shaped by individual characteristics like medical risk and cultural preference operating within structural limits such as hospital closures, staffing shortages, and geographic remoteness [7]. While bypassing can signal systemic access gaps or lower local care quality, it also reflects proactive patient efforts to reach higher-level facilities, a choice often restricted for low-income or Indigenous populations by the burdens of travel distance and cost. Importantly, bypassing to higher-level hospitals is sometimes clinically indicated. High-risk pregnancies (e.g., preeclampsia, preterm labor, fetal anomalies) are appropriately referred to sub-specialty hospitals regardless of patient preference or weather.

Obstetric bypassing is particularly relevant in states with large swaths of rural areas like Montana, where half of all counties are designated as ‘maternity care deserts’ which lack a hospital-based obstetric unit or obstetric providers, requiring long travel distances to maternity care [8,9]. The public health burden of this access gap is severe and is demonstrated by Montana’s 2025 maternal mortality rate of 32.5 per 100,000 live births, ranking 41st of 48 ranked states, and more than double the Healthy People 2030 target of 15.7 deaths per 100,000 births [10]. This access gap is shaped by the financial challenges of providing rural obstetric care, including low reimbursement rates and workforce shortages [11]. Relying solely on the ‘maternity care desert’ designation may be insufficient for planning. Recent optimization analyses suggest that eliminating all deserts would require a prohibitively large number of new facilities, whereas minimizing travel distance offers a more feasible metric for improving access [12].

Recent studies have examined where births occur in Montana and where birthing people choose to deliver [5,13]. This work highlighted that American Indian birthing people living on reservations face the greatest geographic burdens, as they are significantly more likely to bypass local obstetric units and must travel much farther than White residents to access care. Further, Montana has large variations in distances to the nearest facility for different levels of obstetric care. While obstetric units offering specialty and sub-specialty care are situated in population centers, significant geographic barriers remain, as nearly a quarter of ZIP Code Tabulation Areas are over three hours driving time from sub-specialty care and 40% are more than one hour driving time away from any obstetric unit [13,14]. Delivering and receiving prenatal care at non-local facilities offering higher acuity services is an important way that individuals with high-risk pregnancies, such as those involving gestational diabetes, pre-eclampsia, or fetal growth restriction, may receive more risk-appropriate care [6,15,16]. Previous research finds that individuals with pregnancy health-risks are more likely to bypass, but bypassing is still observed among individuals without health risk [5]. Efforts to operationalize such risk-appropriate care in Montana specifically highlight the tension between clinical necessity and the burden of distance for rural patients [17,18].

### 1.2. Knowledge Gap

Despite the importance of these dynamics, studies have not yet focused on the unique environment of sparsely populated mountain states like Montana. A primary weakness in the literature on obstetric health services research is the reliance on static access models. To the best of our knowledge, it is unknown if there are differences in seasonality of births across hospitals of different obstetric levels.

Furthermore, the time-varying nature of obstetric bypassing has not been examined. Montana’s harsh winters bring extreme cold and snow that may impede travel [19] and potentially limit the ability or willingness of patients to bypass their closest facility, even if that facility offers a lower level of care or if higher-level care is preferred. Conversely, milder seasons might facilitate greater bypassing to distant or higher-level facilities. Identifying these interactions is necessary to understand how regional healthcare networks may be impacted by seasonal shifts in patient demand and access.

It remains unclear how these seasonal weather fluctuations influence whether a patient stays local or seeks more distant care. We conceptualize the pathway as follows: seasonal weather conditions alter travel risk and practical accessibility of distant facilities. Birthing people and their care teams weigh this access barrier against the desire for risk-appropriate or preferred care. The resulting facility choice is then observed as a bypassing or non-bypassing event.

### 1.3. Study Objective

This paper examines temporal dynamics of maternal healthcare-seeking behavior in Montana. Specifically, we examine seasonality in birth volume across hospitals of varying levels of care and seasonality in the rate at which people deliver at a non-local obstetric unit.

### 1.4. Hypotheses

- **H1:***Obstetric bypassing rates will decrease during winter months as inclement weather and hazardous driving conditions create physical barriers that impede travel*.
- **H2:***Obstetric bypassing rates will increase during peak birth months (summer)*.

### 1.5. Contributions

This paper makes several contributions to the literature on obstetric health services research. To the best of our knowledge, this paper is the first to examine seasonal differences in birth volume across hospital obstetric levels. This study combines analyses of birth seasonality by hospital level of care with an investigation into the temporal variability of maternal travel for delivery, filling a gap in the literature on rural healthcare utilization. As a rural state with limited and dispersed obstetric services and a high degree of seasonal variability in weather, Montana serves as a useful case study for such analysis. This analysis will provide empirical data to support public health resource planning in regions with extreme seasonal variability.

## 2. Materials and Methods

### 2.1. Research Design

We conducted a retrospective analysis of secondary data (birth records) to estimate seasonal distributions of birth volume and obstetric bypassing rates at hospital-based obstetric units that provide different levels of care in the rural state of Montana. Software analysis was performed using Stata 15 for statistical modeling.

### 2.2. Data Sources

**Figure 1:**
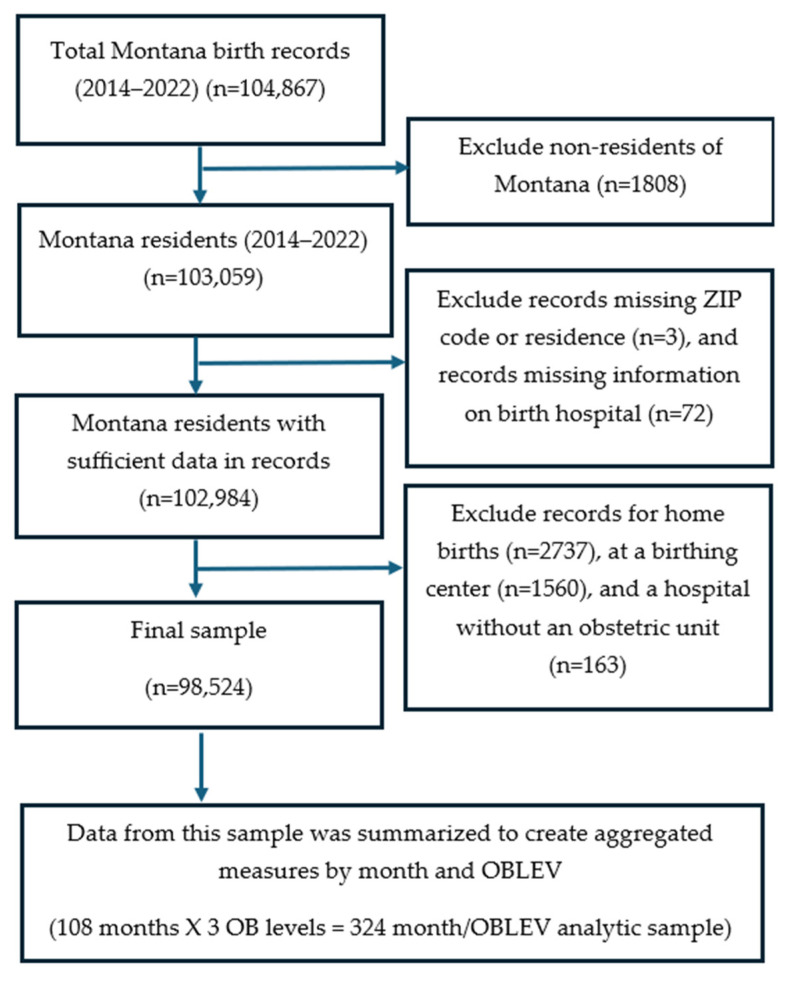
Study population flow chart of sample exclusions.

Data on births came from Montana birth records (2014–2022; *n* = 104,867). Non-residents of Montana (*n* = 1808), records missing the birthing person’s ZIP code of residence (*n* = 3), and records missing information on birth hospital (*n* = 72) were excluded, which was required to calculate travel distance and local hospital status. Due to the study’s focus on births at hospital-based obstetric units, records for individuals who gave birth at home (*n* = 2737), at a birthing center (*n* = 1560), or at a hospital without an obstetric unit (*n* = 163) were excluded, resulting in a final analytic sample of 98,524 births (with 27,548 births at obstetric unit level 1 hospitals, 34,879 births at obstetric unit level 2 hospitals, and 36,097 births at obstetric unit level 3 hospitals). Data from individual birth records from this sample were then summarized into a time series of monthly totals for each hospital level of obstetric care (OBLEV; Levels 1, 2, and 3), to create aggregated measures for each month/OBLEV. Note that the birth volume analysis includes all nine years of data (2014–2022) providing 108 months, while the obstetric bypassing analysis excluded December 2022 to maintain the integrity of the seasonal coding (where December is grouped with the following year’s winter, “Winter 2023”). For the birth volume analysis, this resulted in 108 months of data for each of the three OBLEV levels, yielding a total analytic sample size of *n* = 324. For the obstetric bypassing analysis, this resulted in 107 months of data for each of the three OBLEV levels, yielding a total analytic sample size of *n* = 321. Figure 1 shows the study population flow chart of sample exclusions. Data on the ZIP code of residence and hospital addresses were used to identify births that were bypassed births, defined as births delivered at a non-local hospital. “Local” OB unit(s) are defined as any OB unit(s) within 15 miles of the birthing person’s closest OB unit [5,6]. Driving distances were calculated using ArcGIS Pro 3.1 from the population-weighted centroid of the ZIP code of residence to each hospital in the state (including all hospitals that reported at least one birth during the period of study). This information was used to determine whether births at each hospital were local or non-local.

Data on hospital characteristics came from 2014–2022 American Hospital Association (AHA) surveys. Hospital level of obstetric care (OBLEV) included: Level 1 (basic care, offering services for uncomplicated cases), Level 2 (specialty care, services for all uncomplicated and most complicated cases), and Level 3 (sub-specialty care, services for all complicated cases).

Monthly average precipitation (at the month-county level, measured in inches per month) and temperature data (at the month-county level, measured in degrees Fahrenheit) were obtained from the National Centers for Environmental Information [20]. For each OBLEV, we calculated the average temperature and precipitation, weighting the county-level data by the number of hospital facilities in that county. These variables were standardized to have a mean of zero and a standard deviation of one.

### 2.3. Birth Volume-Related Measures

**Figure A1:**
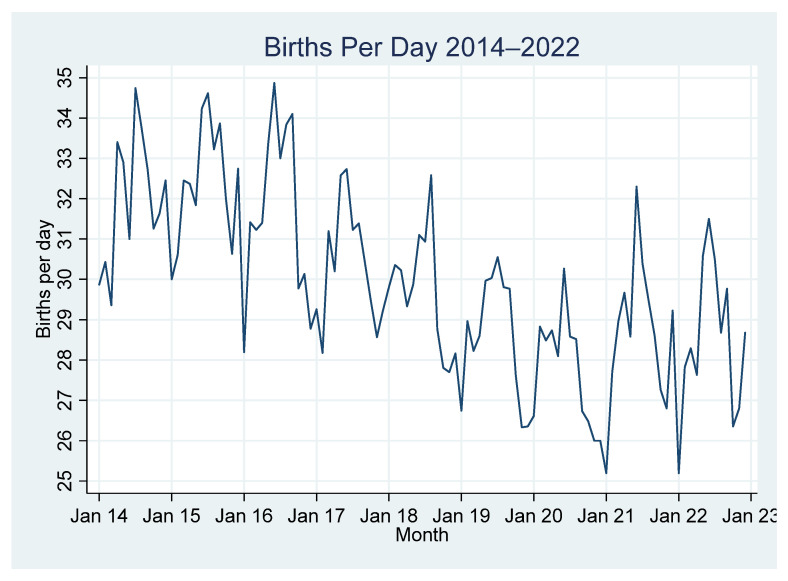
Monthly average births/day for the years 2014 through 2022.

**Figure A2:**
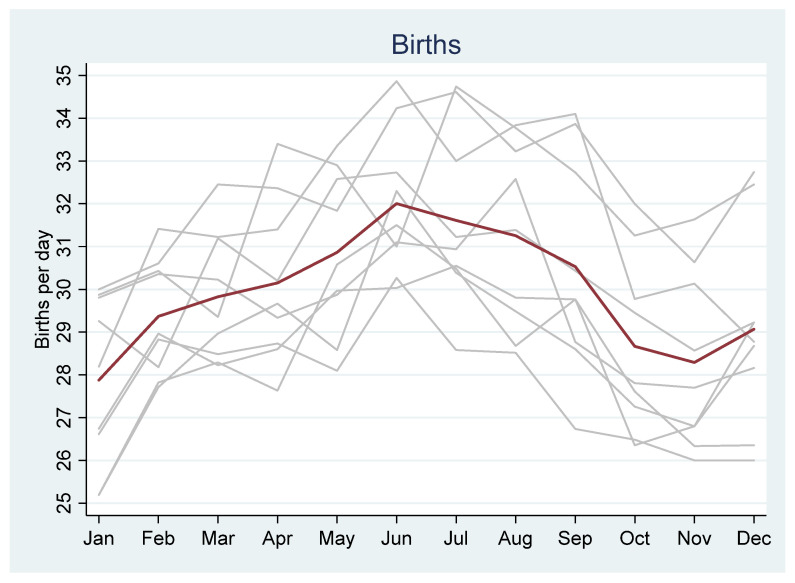
Births/day for each month (gray lines for the years 2014 through 2022; red line for overall average).

Individual birth records were summarized into a time series of monthly totals for each OBLEV (Levels 1, 2, and 3). The variable births/day was computed for each month by dividing the number of births in each month by the days in the month for each of the 108 months of data. The monthly data were stratified by OBLEV (Levels 1, 2, and 3). Data are characterized by a decreasing monthly births/day trend over the time horizon examined for each OBLEV (Figure A1). Figure A2 overlays the data across years and shows visually that overall, births/day exhibits seasonality.

To capture the seasonality of the data, we calculated a ratio of the observed-to-expected births/day for each month in the study period. Data were converted into a centered twelve-month moving average to capture long-term trends. An observed-to-expected births/day ratio was calculated by dividing births/day by the moving average. This approach enables us to adjust for the temporal trend in births prior to estimating the seasonal association with birth volume. A ratio value less than one can be interpreted as being below average, and a ratio greater than one is above average.

### 2.4. Bypassing-Related Measures

In the analyses examining the seasonality of bypassing, to increase sample size and improve parsimony in models, we grouped months into four season categories (Winter: December–February; Spring: March–May; Summer: June–August; Autumn: September–November; we coded December of year X as Winter of year X + 1) and calculated bypassing rates for each season.

Individual birth records were summarized into a time series of seasonal totals for each obstetric unit level of care. Two variables were created, capturing the seasonal bypassing-to rate and the bypassing-from rate.

#### 2.4.1. Bypassing-To Rate Measure

Conceptually, the bypassing-to rate measures the rate of patient inflow, defined as the proportion of total births at a specific level of care contributed by non-local patients. When calculating the bypassing-to rate, for each season the number of births that occurred at a non-local facility of that OBLEV was divided by the total number of births that occurred in the season at all hospitals of that OBLEV. In this operationalization strategy, the unit of analysis is the year-season of birth, with separate observations for each OBLEV-level, representing births bypassed to a hospital of a specific level of care in a given year-season.

(1) $$\begin{array}{c} N_{t}:\;\mathrm{Total}\;\mathrm{births}\;\mathrm{at}\;\mathrm{hospitals}\;\mathrm{of}\;\mathrm{OBLEV}\;X\;\mathrm{by}\;\mathrm{non-local}\;\mathrm{patients}\\ D_{t}:\;\mathrm{Total}\;\mathrm{births}\;(\mathrm{local}\;+\;\mathrm{non-local})\;\mathrm{occurring}\;\mathrm{at}\;\mathrm{all}\;\mathrm{hospitals}\;\mathrm{of}\;\mathrm{OBLEV}\;X \end{array}$$

To adjust for any trends in all bypassing-to variables over time, a centered four-period (with periods corresponding to the four seasons) moving average (MA) was computed for each OBLEV and season *t* by weighting the sums of the above quantities as follows:

(2) $$\begin{array}{c} {\mathrm{MA}}_{\mathrm{numerator}}(t)\;=\;1(N_{t-2})\;+\;2(N_{t-1}\;+\;N_{t}\;+\;N_{t+1})\;+\;1(N_{t+2})\\ {\mathrm{MA}}_{\mathrm{denominator}}(t)\;=\;1(D_{t-2})\;+\;2(D_{t-1}\;+\;D_{t}\;+\;D_{t+1})\;+\;1(D_{t+2})\\ MA\left(t\right)=\frac{MA_{numerator}(t)}{MA_{denominator}(t)} \end{array}$$

Finally, an observed-to-expected bypassing-to ratio was calculated for each season and each year by dividing bypassing-to rate by its respective moving average.

#### 2.4.2. Bypassing-From Rate Measure

Conversely, the bypassing-from rate measures the rate of patient outflow, defined as the proportion of potential patients who bypass their nearest hospital to deliver elsewhere. When calculating the bypassing-from rate, for each season the number of births that occurred in which the person bypassed away from their local hospital of that OBLEV was divided by the total number of births that occurred for which that OBLEV was the closest available level of care. The unit of analysis is the year-season of birth, with separate observations for each OBLEV-level, representing births bypassed away from a hospital of a specific level of care in a given year-season.

(3) $$\begin{array}{c} N_{t}:\;\mathrm{Total}\;\mathrm{births}\;\mathrm{where}\;\mathrm{the}\;\mathrm{patient}’s\;\mathrm{closest}\;\mathrm{hospitals}\;\mathrm{is}\;\mathrm{OBLEV}\;X\;\mathrm{but}\;\mathrm{the}\;\mathrm{patient}\;\\ \mathrm{bypassed}\;\mathrm{it}\;\mathrm{to}\;\mathrm{deliver}\;\mathrm{at}\;a\;\mathrm{different}\;\mathrm{hospital}\;\mathrm{in}\;\mathrm{season}\;t\\ D_{t}:\;\mathrm{Total}\;\mathrm{births}\;\mathrm{occurring}\;\mathrm{at}\;\mathrm{all}\;\mathrm{hospitals}\;\mathrm{whose}\;\mathrm{closest}\;\mathrm{hospital}\;\mathrm{was}\;\mathrm{OBLEV}\;X\;\\ \mathrm{regardless}\;\mathrm{of}\;\mathrm{delivery}\;\mathrm{location} \end{array}$$

A centered four-period (with periods corresponding to the four seasons) moving average was computed to capture any trends by applying Equation (2). Finally, an observed-to-expected bypassing-from ratio was calculated for each season and each year by dividing bypassing-from rate by its respective moving average. This approach enables us to adjust for the temporal trend in births prior to estimating the seasonal association with bypassing.

### 2.5. Statistical Analysis

To quantify these trends, OLS regression models were estimated for each OBLEV with births/day as the dependent variable and the monthly date as the independent variable. Temporal analyses of bypassing trends over time were also conducted using OLS regression models. Monthly and seasonal differences in observed-to-expected ratios were evaluated using OLS regression with calendar month (or season) indicator variables, with standard errors clustered by year to account for within-year temporal dependence. Bonferroni adjustments were applied for pairwise comparisons across months (or seasons). We estimated a panel fixed effects model (specifically, a within-estimator) using Stata’s xtreg command to examine factors associated with O-E bypassing ratios. To examine unit-specific heterogeneous effects, we interacted our primary independent variable (season) with the panel indicator (OBLEV). Standard errors are clustered at the OBLEV level to account for within-unit serial correlation. The statistical analyses were performed using Stata.

Post-estimation diagnostics were used to validate panel fixed effects model assumptions as follows. Homoscedasticity was confirmed using the Breusch–Pagan test, which yielded non-significance. Diagnostic testing for the normality of residuals was conducted using the Shapiro–Wilk test (W = 0.960, *p* < 0.001). While the formal test indicates a departure from perfect normality, visual inspection of the kernel density plots confirms that the residuals are approximately bell-shaped and symmetrically distributed. Given that panel fixed effects models are robust to non-normality in samples of this size (*n* = 321), and that our primary focus is on the consistency of seasonal trends in Montana obstetric bypassing, these diagnostics confirm the reliability of our coefficient estimates as Best Linear Unbiased Estimators (BLUE). To test for multicollinearity, we computed the variance inflation factor (VIF) for each model (run as OLS models). Across all models, the largest VIF value was the ‘Summer’ indicator in model 4 which was slightly above 10 (10.76), and this is attributed to the inclusion of interaction terms between season and obstetric level of care. In all other models, all predictors, including weather variables, remained within acceptable limits.

## 3. Results

### 3.1. Birth Volume Seasonality Analysis

The birth volume analysis confirms a downward trend across all hospital levels in Montana from 2014–2022 (Table A1). OLS regression models, using the month as the independent variable, show that the full sample experienced a decline of −0.048 births/day per month. (*p* < 0.001). OBLEV 1 hospitals experienced the steepest decline (−0.021 births/day per month; *p* < 0.001). Comparatively, OBLEV 2 and 3 hospitals showed a rate of decline at −0.011 births/day per month (*p* < 0.001) and −0.016 births/day per month (*p* < 0.001), respectively.

**Figure 2:**
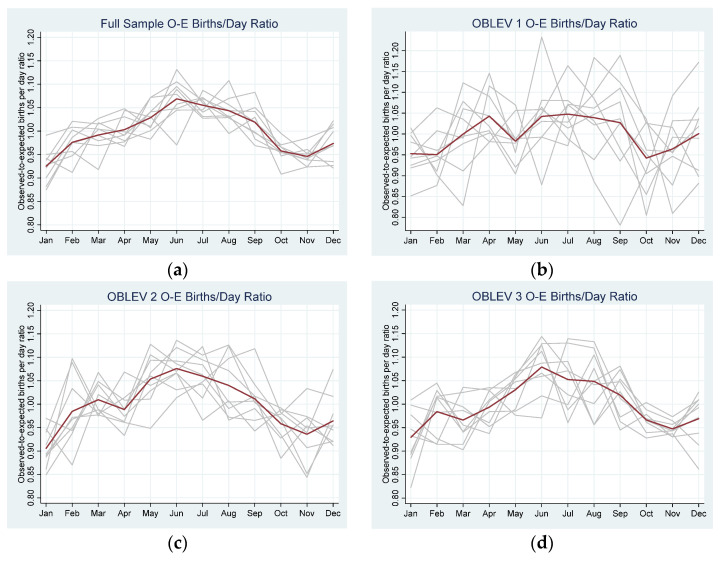
Monthly observed-to-expected (O-E) births/day ratio for the overall sample and each OB level: (**a**) Overall sample; (**b**) OB Level 1; (**c**) OB Level 2 sample; (**d**) OB Level 3 sample. Note: The red line shows the average O-E births/day ratio across all 9 years for each month, and the gray lines show the O-E births/day ratio for each month for each of the 9 years.

Figure 2 shows the observed-to-expected births/day ratio for the full sample and each OBLEV overlaid across years. Seasonality in the data is apparent given the consistent ratios across years for the full sample, Level 2, and Level 3. The variability of Level 1 ratios across years provides less evidence of seasonality. The above-average period for the overall sample lasts from May through September (peaking in June) and the below average period lasts from October through February (reaching a low in January). Figure 2b shows the observed-to-expected births/day ratio at Level 1 hospitals. Notice the higher variability across years compared to the overall sample (Figure 2a). Births/day at Levels 2 and 3 (Figure 2c,d) have slightly higher seasonal peaks in June than the overall sample.

Pooled OLS estimates shown in Table 1 examined the statistically significant differences in the observed-to-expected births/day ratio for each calendar month for the overall sample, and the OBLEV 1, 2, and 3 samples. The overall sample shows a seasonal peak in the summer. Specifically, O-E births/day ratio in June (1.069), July (1.055) and August (1.043) were significantly higher than the January trough and several autumn/winter months (*p* < 0.05, see Table 1). The seasonal low in January (O-E births/day ratio 0.925, representing approximately 7.5% fewer births/day than the annual average) was found to be significantly lower than nearly all other months (February through October and December, *p* < 0.05), providing evidence of the Winter trough. Given the overall average of thirty births/day for the overall sample, this deviation represents 2.25 fewer births/day (around 68 births per month) at the state level. Table 1 shows that many differences are statistically significant across months for the overall sample. The OBLEV 1 sample does not show statistically significant differences in observed-to-expected ratios. The winter trough of lower O-E births/day in January (0.906) relative to Spring and Summer months was observed at OBLEV 2 hospitals (*p* < 0.05). For OBLEV 3 hospitals, the June peak (1.079) was higher than the ratios in March and November (*p* < 0.05).

To examine the strength of the relationship between the dependent variable observed-to-expected births/day ratio and the set of calendar month indicator variables, we conducted supplementary regression analyses (Table A2). Results show that 66.39% of the variance was explained by the monthly indicator variables in the overall model (R^2^ = 66.39%), and 20.57%, 52.24%, and 48.08% of the variance was explained by the monthly indicator variables in the OBLEV 1, 2, and 3 models, respectively. The presence of seasonality of births is clear for the overall sample, and for OBLEV 2 and 3 samples, but it is less clear for the OBLEV 1 sample. Looking closely at the pattern of observed-to-expected births/day ratios over the course of a calendar year, a seasonal pattern emerges with a lower ratio during Winter months (December–February), a slightly higher ratio in the Autumn (September–November), the ratio again slightly higher and hovering around 1 in the Spring (March–May), and the highest ratio of observed-to-expected births/day during Summer months (June–August). Given this seasonal pattern, in the next set of analyses examining the seasonality of bypassing, to increase sample size and improve parsimony in models, we grouped months into four season categories (Winter: December–February; Spring: March–May; Summer: June–August; Autumn: September–November; we coded December of year X as Winter of year X + 1), and calculated bypassing rates for each season.

### 3.2. Bypassing Seasonality Analysis

The overall average bypassing-to rate for OBLEV 1 was 11.6%; OBLEV 2 was 8.2%; OBLEV 3 was 16.8%. The overall average bypassing-from rate for OBLEV 1 was 31.1%; OBLEV 2 was 4.8%; OBLEV 3 was 0.9%. Temporal analysis of the overall bypassing-to rate showed an upward trend over the study period. The overall bypassing-to rate increased by 0.022% per month (Table A3; *p* < 0.001). However, the trend is driven by an increase in bypassing toward OBLEV 3 hospitals, which increased at a rate of 0.062% per month (95% confidence interval [0.00055, 0.00068]; *p* < 0.001). There were no significant trends observed for OBLEV 2 hospitals (*p* = 0.235) and only a marginally significant downward trend for OBLEV 1 hospitals (*p* = 0.078).

**Figure 3:**
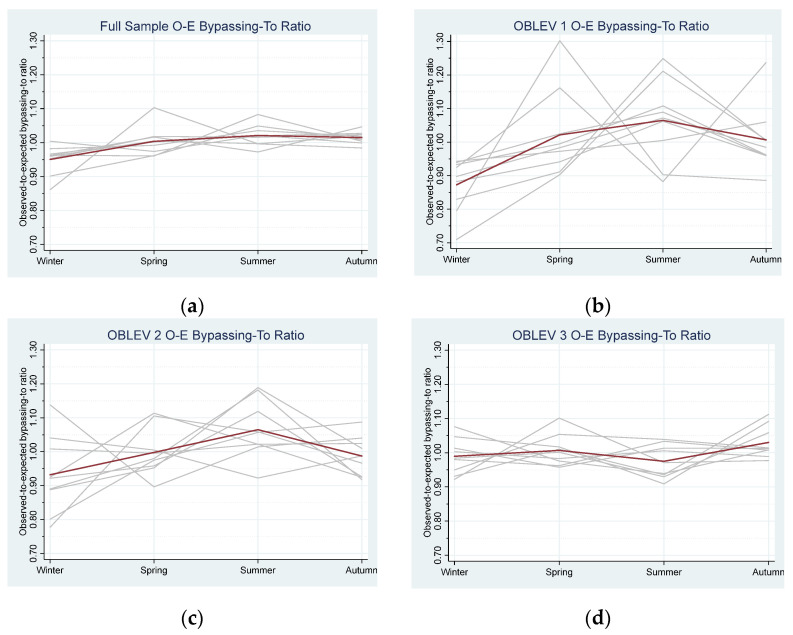
Seasonal observed-to-expected (O-E) bypassing-to ratio for the overall sample and each OB level: (**a**) Overall sample; (**b**) OB Level 1; (**c**); OB Level 2 sample; (**d**) OB Level 3 sample. Note: The red line shows the average O-E bypassing-to ratio across all 9 years for each season, and the gray lines show the O-E bypassing-to ratio for each season for each of the 9 years.

**Figure A3:**
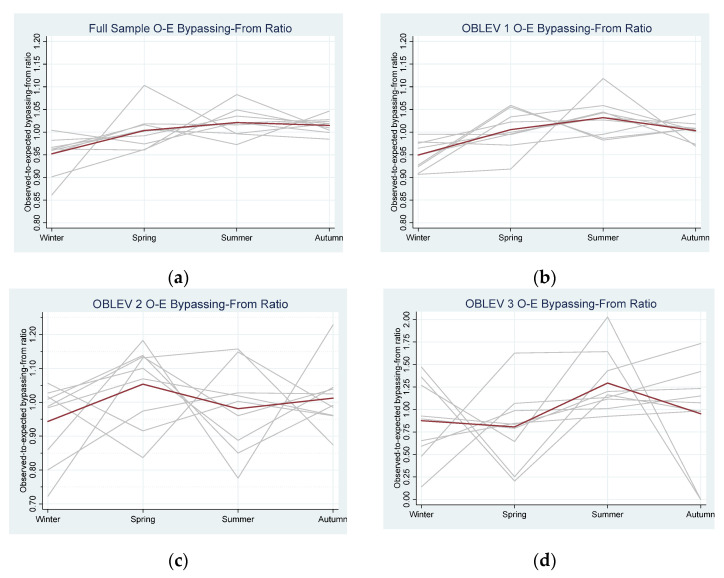
Seasonal observed-to-expected (O-E) bypassing-from ratio for the overall sample and each OB level: (**a**) Overall sample; (**b**) OB Level 1; (**c**) OB Level 2 sample; (**d**) OB Level 3 sample. Note: The red line shows the average O-E bypassing-from ratio across all 9 years for each season, and the gray lines show the O-E bypassing-from ratio for each season for each of the 9 years.

The average bypassing-to rate for each season is shown in Figure 3, while the bypassing-from rate is presented in Figure A3. There is a noticeable low season during winter for the overall sample (O-E bypassing-to ratio of 0.950, representing approximately a 5% reduction relative to the annual average, see Figure 3a). When focusing on bypassing to OBLEV 1, there is an even starker low season (O-E ratio of 0.8726, a 12% reduction in non-local patient inflow at OBLEV 1 during Winter, see Figure 3b).

Pooled OLS results (Table 2) confirm that there is a statistically significant seasonal trough in winter observed-to-expected (O-E) bypass-to ratios for the overall sample and the OBLEV 1 sample. There is less evidence of seasonality in the bypassing-to rate for OBLEV 2 and 3 hospitals, as their O-E ratios stay within narrower ranges of 0.933–1.065 and 0.975–1.030 respectively. Supplementary regression analyses predicting the O-E bypassing-to ratio (see Table A4) indicate that season helps to explain a substantial proportion of the variation in bypassing-to, with an R^2^ of 39.28% for the overall sample, 30.87% for OBLEV 1 sample, 25.36% for the OBLEV 2 sample, and 16.71% for the OBLEV 3 sample. This suggests that, particularly for the overall sample and OBLEV 1 hospitals, season is a strong predictor of variation in rates of bypassing toward these hospitals. For OBLEV 1 hospitals, bypassing-to rates are lowest in Winter.

Turning to bypassing-from rates, we observe higher bypassing-from rates for OBLEV 1 hospitals across all seasons (29–32%), compared to OBLEV 2 (4–5%) and OBLEV 3 (0.7–1.2%), with bypassing-from rates lowest in the winter for all OBLEVs. Pooled OLS results comparing average O-E bypassing-from ratios highlight that there is a statistically significant trough in bypassing away from OBLEV 1 in the winter. Given the low rates of bypassing-from OBLEV 3, O-E bypassing-from ratios are not very meaningful. Supplementary regression analyses predicting the O-E bypassing-from ratio (see Table A5) indicate that season helps to explain a substantial proportion of the variation in bypassing-from OBLEV 1 hospitals (R^2^ of 43.3%), and for the overall sample (R^2^ = 39.3%), and a lesser amount for the other samples (12.0% for the OBLEV 2 sample, and 15.7% for the OBLEV 3 sample).

To formally test whether the seasonality in bypassing-to and bypassing-from differs across OBLEVs, we ran a series of panel fixed effects models predicting the O-E bypassing-to (and -from) ratios including both season and OBLEV, and their interaction (Table 3). Accounting for these fixed effects controls for all unobserved, time-invariant characteristics of our panel variable, OBLEV. Therefore, the main effects of OBLEV are already adjusted for (and are omitted from output). By including the interaction effect between OBLEV and seasonality we were able to test if hospital OBLEV moderates the association between season and bypassing; is the Winter trough in bypassing stronger at hospitals with lower OBLEVs?

**Figure A4:**
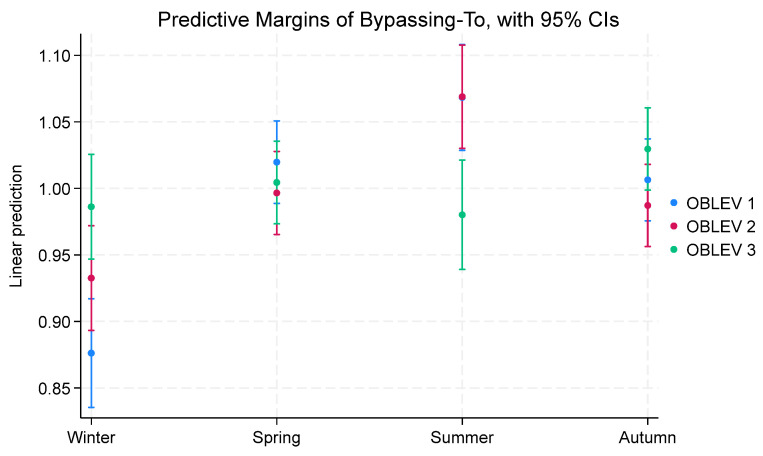
Predictive margins for predicting O-E bypassing-to by Season and OBLEV, as estimated in Table 3, Model 4.

Accounting for interaction effects, we observe that in Winter, OBLEV 2 and 3 hospitals have a significantly higher O-E bypassing-to ratio compared to OBLEV 1 hospitals (Model 2). Further, interaction terms indicate that while Spring generally has higher O-E bypassing-to ratios compared to Winter, the increase is less pronounced for OBLEV 2 and 3 hospitals compared to OBLEV 1. Similarly, the interaction term between Summer and OBLEV 3 is strong, negative, and highly significant, meaning that while OBLEV 1 hospitals see a large increase in bypassing-to ratios in the summer, OBLEV 3 hospitals see smaller O-E ratios. To ease interpretation of interaction effects, post-estimation linear predictive margins of the O-E bypassing-to ratio is visualized in Figure A4.

Now we turn to the fixed effects models for bypassing-from ratios. Compared to Winter, the Summer season is associated with a highly significant increase in the O-E bypassing-from ratio, suggesting hospitals lose significantly more birthing patients than expected in Summer compared to the Winter. Including OBLEV interactions in the model (Model 6), we see a large and significant interaction between Summer and OBLEV 3, while the main effect for Summer is no longer significant, suggesting that only OBLEV 3 hospitals lose substantially more nearby birthing patients than expected during the Summer.

**Figure A5:**
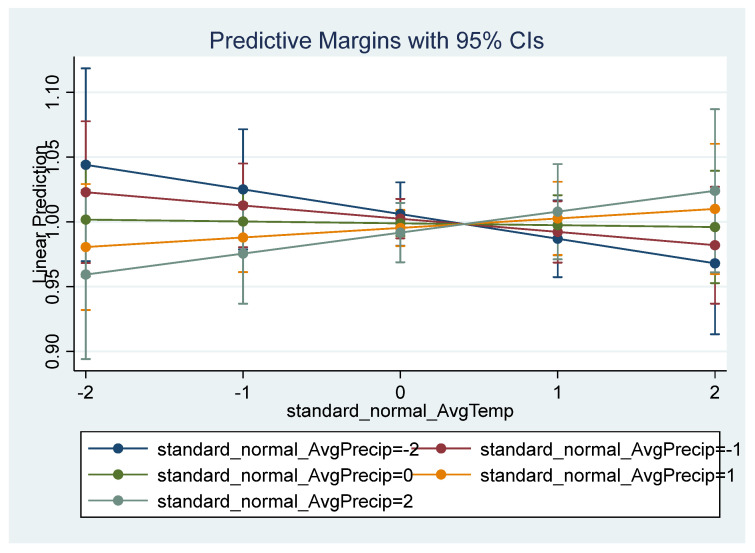
Predictive margins for predicting O-E bypassing-to by average monthly temperature and precipitation, as estimated in Table 3, Model 3.

We also ran regression models including weather as a predictor, to test if more inclement weather during winter months helped to explain the trough in bypassing during Winter months (Table 3, Models 3 and 4). This included an interaction between average monthly temperature and average monthly precipitation to capture different weather conditions (i.e., low temperature and high precipitation is characteristic of snowstorms). Results point to a moderately significant interaction (*p* < 0.1) between temperature and precipitation. To ease interpretation, post-estimation linear predictive margins were calculated (Figure A5). This figure shows the predicted O-E bypassing-to ratio on the vertical axis as a function of standardized average temperature, conditional on different levels of standardized average precipitation. These results indicate a moderately significant interaction between average precipitation and average temperature on the O-E bypassing-to ratio. At average temperature (standardized average temperature = 0) the predicted O-E bypassing-to ratio is similar across average precipitation levels. At lower temperatures (e.g., standardized temperature = −2) higher levels of standardized precipitation are associated with lower predicted O-E bypassing-to ratios. At higher temperatures (e.g., standardized average temperature = 2) the pattern reverses. These results reveal an association where the O-E bypassing-to ratio fluctuates alongside monthly climate averages. Specifically, lower O-E bypassing-to ratios are associated with periods characterized by low average temperature and high precipitation, while higher O-E bypassing-to ratios correspond to months with high average temperature and high precipitation.

## 4. Discussion

We organize our discussion of the results at three levels: descriptive findings on the seasonality of volume and flows, mechanistic hypotheses, and systematic implications for rural healthcare planning that follow from the descriptive patterns.

### 4.1. Descriptive Findings on the Seasonality of Birth Volume and Flows

Our findings indicate that Montana experiences seasonal birth volume fluctuations that are distinct from national trends. Unlike the late-summer peaks typically observed in the U.S. [1], Montana’s birth volume surges earlier, with pronounced peaks in June and troughs in winter. Prior research confirms that birth seasonality shifts with latitude, predicting earlier spring/summer peaks for northern populations compared to the autumn peaks typical of the southern U.S., and our findings are consistent with this latitudinal gradient [3]. This seasonality is more pronounced at specialty (Level 2) and sub-specialty hospitals (Level 3), while basic care (Level 1) hospitals exhibit comparatively milder seasonal variation, a finding that, to our knowledge, has not previously been documented across hospital obstetric levels.

Over the study period, there is a decreasing trend in birth volume across all hospital levels (−0.048 births/day per month), and the impact is not uniform. Our results suggest that decreasing birth volume over time in Montana disproportionately impacted OBLEV 1 hospitals, where birth volume is decreasing faster than at Level 2 and 3 hospitals. This downward trend aligns with recent evidence suggesting that rural hospitals face unique financial challenges that make sustaining obstetric units difficult [11], contributing to recent obstetric unit closures in the rural US [21,22,23] which can ultimately worsen maternal and infant birth outcomes in rural places [24].

This demographic trend can also be contextualized with the finding that there has been an increase in bypassing toward hospitals that offer sub-specialty care (Level 3), with a greater share of births at Level 3 hospitals including those that were from non-local people. Our trend analysis (Table A3) provides evidence to support the observation that bypassing toward OBLEV 3 hospitals significantly increased over time, with a monthly increase of 0.062% (*p* < 0.001). This represents a cumulative increase of approximately 6.6% in the OBLEV 3 bypassing-to rate across the 107-month study period. Given the 36,097 births that occurred at OBLEV 3 hospitals in our study over 9 years, for an average of 4010 OBLEV 3 births/year, this 6.6% increase represents an additional several hundred (~265) bypassed births at OBLEV 3 hospitals. However, this increasing reliance on distant care warrants caution, since recent evidence suggests that rural patients who bypass local care for urban hospitals may face higher rates of severe maternal morbidity and mortality [25].

During the Spring, Summer, and Autumn seasons there is a significant increase in people bypassing away from Level 1 hospitals (more births leave than expected within the service area). Summer is also a time when we observed a higher-than-expected increase in people who live in Level 3 service areas bypassing to other hospitals.

To contextualize the scale of bypassing away from OBLEV 1 hospitals, we conducted supplemental analyses to estimate the potential increase in birth volume at OBLEV 1 hospitals if more people delivered at their nearest facility. In our dataset, over 10,000 births bypassed their closest hospital (which was OBLEV 1) to deliver at an OBLEV 2 (4224 births) or OBLEV 3 (6259 births) hospital. If all people whose closest unit was OBLEV 1 had delivered there, the collective average births per day at these units would increase from 7.06 (baseline) to 10.25—a 45.19% increase in volume. While this analysis ignores the crucial factor of risk-appropriate care, it provides a reasonable upper bound on potential birth volume at OBLEV 1 hospitals and highlights the potential benefits of attracting patients from their local service areas for capacity planning.

### 4.2. Mechanistic Hypotheses

The seasonal patterns documented are consistent with several plausible mechanisms, though our data do not permit us to confirm them directly.

Results indicate that there is less pronounced seasonality in bypassing rates toward the more complex OB care (Levels 2 and 3); the relative stability of bypassing to complex care across seasons is consistent with two non-exclusive explanations. First, clinically indicated referrals to sub-specialty care proceed regardless of season or weather. Second, patients with complex pregnancies may plan care well in advance, reducing sensitivity to weather at the time of delivery. We cannot distinguish these mechanisms with the available data. Generally, results point to less seasonal variability in bypassing-to hospitals offering more complex care (Levels 2 and 3), while hospitals offering basic care (level 1) experience a larger summer peak.

The higher bypassing-to rates in the summertime at OBLEV 1 hospitals might also be due to increased traveling during the summer. As people drive more in the summer, often to more rural or dispersed places for outdoor recreation, this may lead people to bypass (intentionally or unintentionally) at higher rates in more rural areas where these Level 1 hospitals are located.

Models incorporating weather conditions provide some support for this interpretation. While our results show that bypassing is lowest during cold and high-precipitation months, this finding is consistent with the hypothesis that patients engage in ex-ante risk aversion, planning local delivery to avoid uncertain winter travel. The county-level monthly averages we use as weather correlates do not capture route-specific road conditions, snowfall accumulation, or storm events, so our weather variables should be interpreted as seasonal climate correlates rather than direct measures of travel impedance. Direct surveys of rural patients are required to validate this proposed mechanism. Practically, the Winter trough in bypassing to OBLEV 1 hospitals (O-E bypassing-to ratio 0.879) translates to a roughly 12% reduction in non-local patient inflow during those Winter months, which may result in pressure on the hospital system. Patients may factor the likelihood of adverse weather into their birth facility choice, opting for local care during months of high weather volatility to avoid difficult travel. Such advanced planning is critical in places like Montana, where harsh winters and vast distances frequently compromise timely transport [17]. Thus, the influence of specific winter storms is largely absorbed into the baseline decision to stay local.

Our models also indicate that bypassing is highest during warm, high-precipitation months. One interpretation is that such conditions in Montana, a climate characterized by low humidity and one prone to summer wildfire smoke, may indicate periods of lower fire risk and better visibility, when longer-distance travel is more feasible. However, this remains speculative. The association could also reflect unmeasured seasonal factors (e.g., summer recreation travel) rather than a direct relationship between weather and access.

### 4.3. Systemic Implications for Rural Healthcare Planning

Several implications follow directly from the descriptive patterns, independent of the mechanisms that drive them.

Policy efforts to sustain rural obstetric units must distinguish between volume loss due to shifting demographics (e.g., aging populations in rural areas) versus volume loss due to bypass behavior, as these drivers require distinct retention strategies.

Seasonal volatility in patient flows carries implications for the implementation of care standardization protocols. In the wintertime, hospitals tend to retain more patients than expected in their service area (O-E bypassing-from ratio < 1). This is especially the case for smaller hospitals offering only basic care, many of which are located in rural areas where wintertime travel may be more difficult. Thus, they must be operationally prepared to deliver standardized care to a potentially more diverse risk pool. Our results suggest that clinical risk management strategies at those smaller hospitals should ensure that standardization of care, facilitated by evidence-based tools like Care Bundles [26], remains reliable throughout the year.

Seasonal fluctuations in bypassing and higher local hospital utilization during winter months indicate a clear need for an integrated regionalized system in Montana. Effective risk-appropriate care depends on a shared and accurate understanding of facility capabilities, which is often inconsistent across rural healthcare networks [18]. Furthermore, the seasonal travel constraints identified in this study are worsened by the decentralized nature of the state’s transport system, and this lack of coordination is a significant barrier to securing timely care during obstetric emergencies [17]. Establishing a centralized statewide transfer process and formalizing rural-specific care designations would help ensure that the seasonal peaks in birth volume are better managed within the regional care network.

### 4.4. Limitations

These findings should be interpreted in light of certain limitations. First, as a retrospective analysis of birth records, we lack clinical data on the specific decision-making process behind each bypass event. We cannot distinguish whether a bypass was driven by medical necessity (physician referral) or patient preference, nor can we observe the specific timing of the decision (e.g., planned months in advance versus an acute reaction). Furthermore, while our weather models suggest risk aversion plays a role in these decisions, the study lacks direct survey data on patient perceptions of travel risk to validate this proposed mechanism.

A second set of limitations involves the scope of the variables analyzed. While we observed seasonal fluctuations in birth volume and bypassing behavior, this study does not link these temporal shifts directly with maternal and neonatal health outcomes. Also, the study lacks individual-level baseline characteristics, such as education, income, and marital status, which are known to create disparities in healthcare access [27].

Finally, our study relies on specific modeling assumptions. We acknowledge that our use of a 15-mile threshold to define “local” status is a static measure that does not fully capture the variability of travel time in rural mountainous regions. While our results are specific to Montana, these findings may be generalized to other rural states, particularly those that experience similar extreme seasonal fluctuations in travel conditions and geographic barriers [17].

Findings suggest that hospitals offering more complex care (Level 3) are increasingly likely over time to receive bypassed births. However, these hospitals face a challenge in retaining childbirth patients during the Summer, losing significantly more patients than expected compared to their usual rates and compared to other hospital levels. This suggests a potential capacity issue, specialized service availability elsewhere, summer travel for patients, or patient preference shifts that disproportionately affect Level 3 hospitals in the summer. Future research is needed to validate our hypotheses about facility capacity strains and patient risk aversion by evaluating the hospitals’ staffing and bed utilization at a more granular level and directly surveying rural patients about how seasonal weather patterns influence their delivery pattern and risk perception.

## 5. Conclusions

Birth seasonality in Montana deviates from national trends, peaking in June and reaching a trough in January, with greater volatility at hospitals offering specialty (Level 2) and sub-specialty care (Level 3). Obstetric bypassing behavior is also highly seasonal, dropping significantly during winter in a pattern consistent with reduced travel under inclement conditions, though causal attribution requires confirmation from direct travel access measures. Conversely, summer surges in bypassing toward higher-level obstetric units may create seasonal capacity strains at regional centers. Ultimately, understanding these fluctuations is essential for optimizing regional healthcare networks and ensuring consistent access to maternal care in extreme climates.

## Figures and Tables

**Table 1:** Monthly Differences in Observed-to-Expected Births/day Ratios (2014–2022).

Month	Overall		OBLEV 1		OBLEV 2		OBLEV 3	
	Mean	Significant Differences at *p* < 0.05 *	Mean	Significant Differences at *p* < 0.05 *	Mean	Significant Differences at *p* < 0.05 *	Mean	Significant Differences at *p* < 0.05 *
1. January	0.925	<6–8	0.953		0.906	<3, 5–8	0.929	
2. February	0.976	<7	0.950		0.984		0.984	
3. March	0.992	<6	1.000		1.009	>1	0.966	<6
4. April	1.003	>11	1.043		0.987		0.993	
5. May	1.029	>11	0.983		1.054	>1	1.031	>11
6. June	1.069	>1, 3, 11	1.042		1.076	>1	1.079	>3
7. July	1.055	>1, 2, 4, 10–12	1.048		1.060	>1	1.052	
8. August	1.043	>1, 10–11	1.039		1.040	>1	1.049	
9. September	1.019	>11	1.027		1.011		1.019	
10. October	0.957	<3–9; >1	0.942		0.958		0.966	
11. November	0.946	<4–9	0.964		0.936		0.947	<5, 6
12. December	0.974	<7	1.001		0.964		0.969	

Notes: OLS regression with calendar month (or season) indicator variables were used for this analysis, with standard errors clustered by year to account for within-year temporal dependence. Bonferroni adjustments were applied for pairwise comparisons across months using the pwcompare post-estimation command in Stata. Abbreviations: OBLEV, Obstetric level of care. * The numbers listed in this column refer to the chronological month of the year, from the first column. A “<” symbol indicates the current month’s ratio is significantly lower than the months listed. A “>” symbol indicates the current month’s ratio is significantly higher than the months listed.

**Table 2:** Seasonal Differences in Observed-to-Expected Bypassing Ratios (2014–2022).

3	Overall			OBLEV 1			OBLEV 2			OBLEV 3		
	Mean Bypass Rate	Mean O-E Ratio	Significant Differences at *p* < 0.05 *	Mean Bypass Rate	Mean O-E Ratio	Significant Differences at *p* < 0.05 *	Mean Bypass Rate	Mean O-E Ratio	Significant Differences at *p* < 0.05 *	Mean Bypass Rate	Mean O-E Ratio	Significant Differences at *p* < 0.05 *
Bypassing-To												
1. Winter	11.49%	0.952	<3–4	10.23%	0.879	<4	7.59%	0.933		16.49%	0.990	
2. Spring	12.16%	1.004		11.81%	1.022		8.13%	0.998		16.86%	1.007	
3. Summer	12.46%	1.021	>1	12.38%	1.065		8.71%	1.065		16.53%	0.975	
4. Autumn	12.50%	1.015	>1	11.73%	1.007	>1	8.12%	0.987		17.65%	1.030	
Bypassing-From												
1. Winter	11.49%	0.952	<3–4	29.40%	0.949	<3–4	4.56%	0.943		0.79%	0.876	
2. Spring	12.16%	1.004		31.36%	1.005		5.06%	1.054		0.69%	0.806	
3. Summer	12.46%	1.021	>1	32.31%	1.032	>1	4.76%	0.981		1.19%	1.295	
4. Autumn	12.50%	1.015	>1	31.74%	1.003	>1	4.90%	1.012		1.09%	0.953	

Notes: OLS regression with season indicator variables were used for this analysis, with standard errors clustered by year to account for within-year temporal dependence. Bonferroni adjustments were applied for pairwise comparisons across months using the pwcompare post-estimation command in Stata. Abbreviations: OBLEV, Obstetric level of care. * The numbers listed in this column refer to the chronological season of the year, from the first column. A “<” symbol indicates the current season’s ratio is significantly lower than the months listed. A “>” symbol indicates the current season’s ratio is significantly higher than the months listed.

**Table 3:** Factors associated with observed-to-expected (O-E) bypassing ratios. Associations were estimated using Panel Fixed Effects (OLS regression).

	Modeling O-E Bypassing-To Ratio				Modeling O-E Bypassing-From Ratio	
	Model 1	Model 2	Model 3	Model 4	Model 5	Model 6
Season (Winter is ref)						
Spring	0.075 *** (5.49)	0.143 *** (6.38)	0.075 *** (4.02)	0.143 *** (5.62)	0.032 (0.73)	0.056 (0.77)
Summer	0.101 *** (7.33)	0.186 *** (8.30)	0.108 *** (3.57)	0.192 *** (5.58)	0.180 *** (4.01)	0.083 (1.13)
Autumn	0.074 *** (5.37)	0.128 *** (5.71)	0.076 *** (3.95)	0.130 *** (5.01)	0.066 (1.51)	0.054 (0.74)
Season × OBLEV interactions						
Spring # OBLEV 2		−0.077 * (−2.45)		−0.080 * (−2.50)		0.054 (0.52)
Spring # OBLEV 3		−0.126 *** (−3.97)		−0.125 *** (−3.95)		−0.126 (−1.23)
Summer # OBLEV 2		−0.053 + (−1.46)		−0.056 + (−1.75)		−0.045 (−0.44)
Summer # OBLEV 3		−0.201 *** (−6.34)		−0.198 *** (−6.23)		0.336 ** (3.26)
Autumn # OBLEV 2		−0.073 * (−2.31)		−0.076 * (−2.38)		0.015 (0.14)
Autumn # OBLEV 3		−0.088 ** (−2.77)		−0.087 ** (−2.73)		0.023 (0.22)
Average county temperature			−0.001 (−0.13)	−0.002 (−0.16)		
Average county precipitation			−0.004 (−0.65)	−0.001 (−0.29)		
Temp × precipitation interaction			0.009+ (1.62)	0.006 (1.17)		
Constant	0.934 *** (94.79)	0.934 *** (101.29)	0.934 *** (57.05)	0.933 *** (60.71)	0.923 *** (29.34)	0.923 *** (30.73)
R-squared	0.159	0.277	0.166	0.280	0.057	0.157

Notes: + *p* < 0.10, * *p* < 0.05, ** *p* < 0.01, *** *p* < 0.001; “#” denotes an interaction term between the specified variables; The unit of analysis is the OBLEV-month. With nine years of data (2014–2022) providing 108 months, we excluded December 2022 to maintain integrity of the seasonal coding (where December is grouped with the following year’s winter, “Winter 2023”), resulting in 107 months of data for each of the three OBLEV levels, yielding a total number of observations, *n* = 321; *t*-statistics in parentheses; OBLEV is birth hospital for models 1–4, and closest hospital with an OB unit for models 5 and 6.

## Data Availability

The birth certificate data that support the findings of this study are available from Montana Department of Health and Human Services, but restrictions apply to the availability of these data, which were used with permission for the current study, and so are not publicly available. The dataset on Montana weather is available from National Centers for Environmental Information (n.d.) Climate at a Glance County Time Series, accessed through https://www.ncei.noaa.gov/access/monitoring/climate-at-a-glance/county/time-series/MT-111/pcp/1/0/2014-2022, accessed on 3 April 2025. The AHA dataset is available, for a fee, from the American Hospital Association (https://www.ahadata.com/, accessed on 3 April 2025). Restrictions apply to the availability of these data, which were used under license for this study.

## Abbreviations

The following abbreviations are used in this manuscript:

AHA	American Hospital Association
ANOVA	Analysis of variance
O-E	Observed-to-expected
OB	Obstetric
OBLEV	Obstetric level of care
US	United States of America

## Appendix A

**Table A1:** Temporal Trends in Birth Volume by Obstetric Level (2014–2022).

Obstetric Level	Coefficient	95% Confidence Interval	*t*-Statistic
Overall Sample	−0.048 ***	[−0.058, −0.037]	−8.87
OBLEV 1	−0.021 ***	[−0.025, −0.017]	−10.56
OBLEV 2	−0.011 ***	[−0.016, −0.005]	−3.73
OBLEV 3	−0.016 ***	[−0.021, −0.010]	−5.66

Notes: *** *p* < 0.001; *n* = 108; The coefficient estimate represents the change in average births/day for each month. All models were estimated using OLS regression, using births/day as the dependent variable and monthly date as the independent variable.

**Table A2:** OLS Regression Models Predicting Observed-to-Expected (O-E) Births/Day Ratio.

Predictor (January Is Ref)	Overall	OBLEV 1	OBLEV 2	OBLEV 3
	Coefficient (Standard Error)	Coefficient (Standard Error)	Coefficient (Standard Error)	Coefficient (Standard Error)
2. February	0.051 * (0.017)	−0.002 (0.0261)	0.079 * (0.032)	0.055 (0.032)
3. March	0.066 ** (0.017)	0.047 (0.039)	0.104 *** (0.020)	0.037 (0.030)
4. April	0.078 *** (0.015)	0.090 ** (0.025)	0.083 *** (0.016)	0.065 * (0.026)
5. May	0.103 *** (0.020)	0.030 (0.026)	0.148 *** (0.025)	0.102 ** (0.027)
6. June	0.144 *** (0.026)	0.089 + (0.039)	0.170 *** (0.021)	0.150 ** (0.035)
7. July	0.130 *** (0.015)	0.095 ** (0.024)	0.154 *** (0.029)	0.123 ** (0.032)
8. August	0.118 *** (0.011)	0.086 * (0.036)	0.134 *** (0.024)	0.119 ** (0.026)
9. September	0.094 ** (0.022)	0.074 (0.049)	0.106 ** (0.025)	0.090 * (0.035)
10. October	0.032 * (0.011)	−0.011 (0.032)	0.053 ** (0.013)	0.037 (0.018)
11. November	0.021 (0.013)	0.011 (0.0337)	0.030 (0.022)	0.018 (0.020)
12. December	0.048 * (0.018)	0.048 (0.030)	0.058 + (0.025)	0.040 (0.022)
Constant	0.925 *** (0.012)	0.953 *** (0.018)	0.906 *** (0.014)	0.929 *** (0.021)
R-squared	0.6639	0.2057	0.5224	0.4808

Notes: + *p* < 0.10, * *p* < 0.05, ** *p* < 0.01, *** *p* < 0.001; *n* = 108; Standard error in parentheses; Standard errors clustered by year to account for within-year temporal dependence.

**Table A3:** Temporal Trends in Bypassing-To Rate by Obstetric Level (2014–2022).

Obstetric Level	Coefficient	95% Confidence Interval	*t*-Statistic
Overall Sample	0.00022 ***	[0.00019, 0.00024]	18.19
OBLEV 1	−0.00010 +	[−0.00021, 0.00001]	−1.78
OBLEV 2	0.00003	[−0.00002, 0.00008]	1.20
OBLEV 3	0.00062 ***	[0.00055, 0.00068]	18.71

Notes: + *p* < 0.10, *** *p* < 0.001; *n* = 107; The coefficient estimate represents the change in average births/day for each month. All models were estimated using OLS regression, using the seasonal bypassing-to rate mapped to the monthly date index as the dependent variable and monthly date as the independent variable. Therefore, while the unit of analysis is a month, the variance occurs at the seasonal level.

**Table A4:** Pooled OLS Estimates of Season Effects on Observed-to-Expected Bypassing-to Ratio (2014–2022).

	Overall	OBLEV 1	OBLEV 2	OBLEV 3
Predictor (Winter Is Ref)	Coefficient (Standard Error)	Coefficient (Standard Error)	Coefficient (Standard Error)	Coefficient (Standard Error)
Spring	0.052 * (0.22)	0.143 * (0.047)	0.065 (0.050)	0.017 (0.025)
Summer	0.069 ** (0.016)	0.186 ** (0.054)	0.132 (0.058)	−0.015 (0.025)
Autumn	0.063 *** (0.009)	0.128 ** (0.035)	0.055 (0.051)	0.040 (0.020)
Constant	0.952 *** (0.011)	0.879 *** (0.021)	0.933 *** (0.036)	0.990 *** (0.012)
R-squared	0.393	0.309	0.254	0.167

Notes: * *p* < 0.05, ** *p* < 0.01, *** *p* < 0.001; *n* = 107; Standard error in parentheses; Standard errors clustered by year. Reference category is Winter.

**Table A5:** Pooled OLS Estimates of Season Effects on Observed-to-Expected Bypassing-From Ratio (2014–2022).

	Overall	OBLEV 1	OBLEV 2	OBLEV 3
Predictor (Winter Is Ref)	Coefficient (Standard Error)	Coefficient (Standard Error)	Coefficient (Standard Error)	Coefficient (Standard Error)
Spring	0.052 * (0.022)	0.056 ** (0.020)	0.110 (0.052)	−0.070 (0.235)
Summer	0.069 ** (0.016)	0.082 *** (0.017)	0.038 (0.058)	0.418 * (0.152)
Autumn	0.063 *** (0.009)	0.054 *** (0.010)	0.069 (0.048)	0.077 (0.225)
Constant	0.952 *** (0.011)	0.949 *** (0.009)	0.943 *** (0.030)	0.876 *** (0.104)
R-squared	0.393	0.433	0.12	0.157

Notes: * *p* < 0.05, ** *p* < 0.01, *** *p* < 0.001; *n* = 107; standard errors in parentheses; Standard errors clustered by year. Reference category is Winter.
